# Supplementary material for: Biophysical analysis of the plant-specific GIPC sphingolipids reveals multiple modes of membrane regulation
Source: J Biol Chem. 2021 Mar 27;296:100602. doi: 10.1016/j.jbc.2021.100602 (PMC8099651; doi:10.1016/j.jbc.2021.100602)

SUPPLEMENTARY DATA FIGURE LEGENDS

Supplementary data 1

High performance thin layer chromatography (HPTLC) assay of eluted fractions collected during the GIPC purification process (described in Figure 2). #2 refers to the crude extract deposited on the silica column. Fractions containing GIPC without visible contamination of sterols and phospholipids were selected and pooled to make up fraction #3.

Supplementary data 2

High performance anion exchange chromatography coupled with Pulsed Amperometric Detection analysis (HPAEC-PAD) of GIPC-enriched samples shows the monosaccharide content after TFA hydrolysis. Abbreviations are as follows: GlcA: glucuronic acid; Glc: glucose; GlcN: glucosamine; Man: mannose; Gal: galactose; Ara: arabinose; Xyl: xylose; Fuc: fucose; Rha: rhamnose; GalA: galacturonic acid. C, Yariv reactivity test of GIPC-enriched samples to detect arabino-galactan content. No arabino-galactan were detected. 50 ug of each sample (1mg/ml) was deposited in each well, the picture was taken 48 h after initiating the reaction. The data shows of one representative analysis from purification of GIPCs, previously checked by HPTLC and LC-MS

Supplementary data 3

Determining glycan content by HPAE analysis of GIPC-enriched samples. A slight change in sugar amount was seen after 1h, 3h and 4h of TFA hydrolysis. (GlcA: glucuronic acid; Glc: glucose; GlcN: glucosamine; Man: mannose; Gal: galactose; Ara: arabinose; Xyl: xylose; Fuc: fucose; Rha: rhamnose; GalA: galacturonic acid).

Supplementary data 4

LC-MS-based sphingolidomic showing the LCB, gluCER, and GIPC content found in crude extract of cauliflower and leek, and purified Bo-GIPC and Ap-GIPC. Analyses were performed on three independent purifications and expressed as the mean +/- SD.

Supplementary data 5

LC-MS-based sphingolidomic showing the different molecular species found in crude extract of cauliflower and leek, and purified Bo-GIPC and Ap-GIPC according to the LCB or (very long chain) fatty acid (VLC)FA content. Analysis were performed on triplicate of three independent purifications. The mean relative amount of each species was calculated and expressed in percentage of total fatty acid.

Supplementary data 6

Phase-contrast microscopy observations of Nt-GIPC containing liposomes in water at RT, pH 7. A, Liposomes obtained after 3 cycles of freeze in liquid nitrogen and thaw (water bath at 60°C) containing (I) Nt-GIPCs 2mg/ml; (II) Nt-GIPC/ 1,2-dimyristoyl-sn-glycero-3-phosphocholine (DMPC) (4:1 mol/mol) at 2mg/ml; (III) Nt-GIPC/ DMPC (1:1 mol/mol) at 2mg/ml shows crystals; (IV) Nt-GIPC/DMPC molar ratio (1:4 mol/mol) at 2mg/ml forms liposomes of 10µm. (V) HPTLC analysis of lipid mixture confirms the presence of GIPC and DMPC in the liposome mix observed. The higher the GIPC content, the higher the occurrence of crystal formation. GIPCs can form liposomes with phospholipids of short acyl chains when the latter is four-folds more abundant in the mix. Scale bar, 5µm. B, Liposomes of Bo-cauliflower GIPC/POPC/ β-sitosterol (1:1:1 mol/mol) at 1mg/ml in TBS 1X. Clusters as shown in (I) were obtained after three cycles of 20min freezing at -20°C and thawing in a water bath at 60°C. Liposomes in (II) were formed after three cycles of freezing in liquid N_2 and 20 min heating in a water bath at 60°C; no cluster was observed. The type of freeze/thaw determines the size and shape of liposomes formed. Scale bar, 5 µm.

Supplementary table 1

Molecular and fragmentation ions of plant sphingolipids monitored by LC/MS^2.

Supplemental data 1

Bo-GIPC (cauliflower)

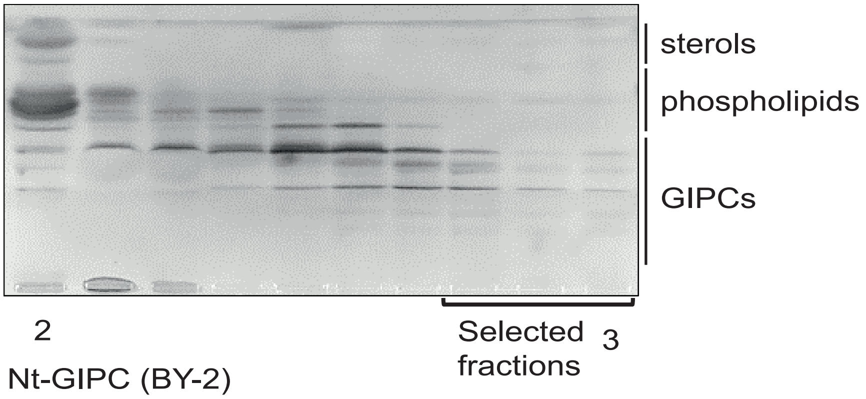

Nt-GIPC (BY-2)

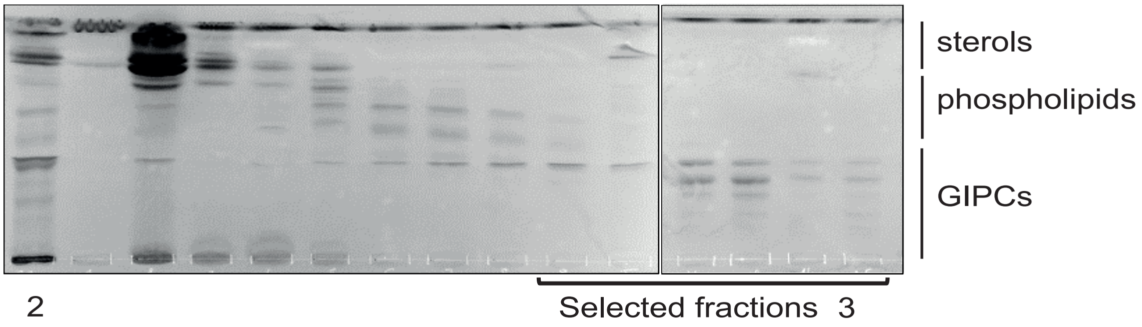

Ap-GIPC (leek)

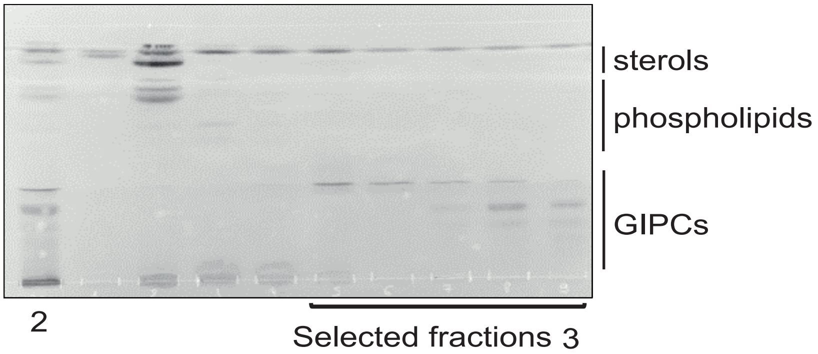

Os-GIPC (rice)

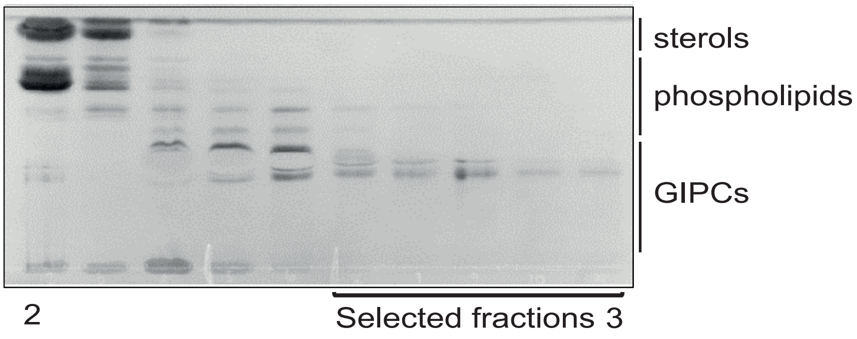

Supplemental data 2

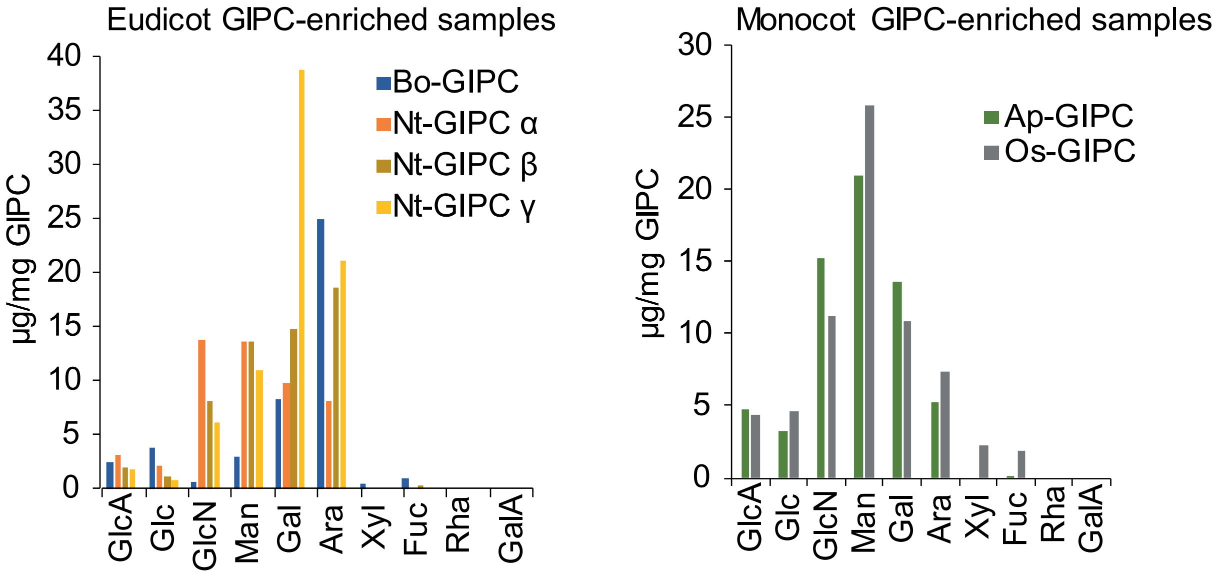

Supplemental data 3

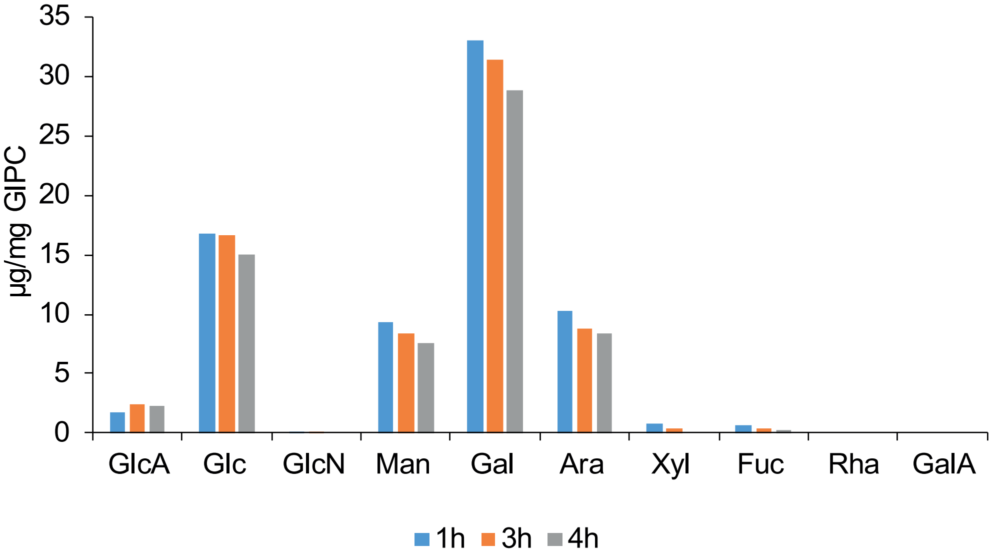

Supplemental data 4

Mean percentage of sphingolipid

Cauliflower

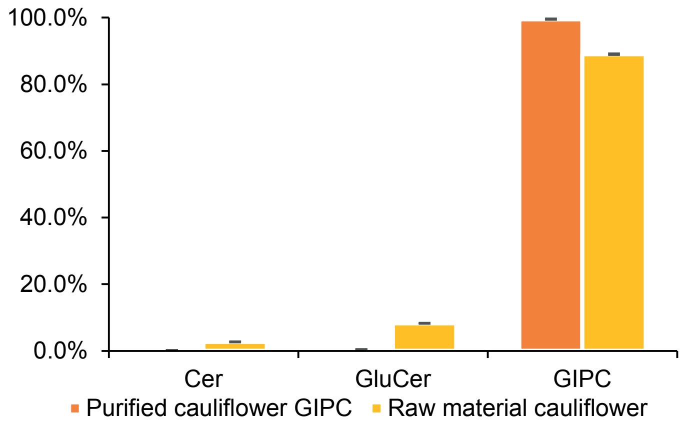

Leek

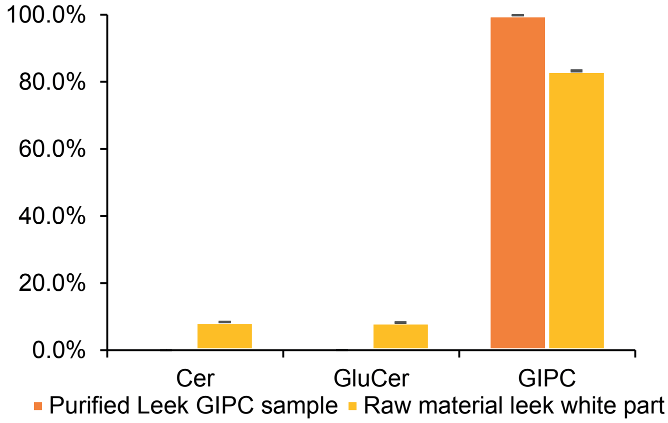

Supplemental data 5

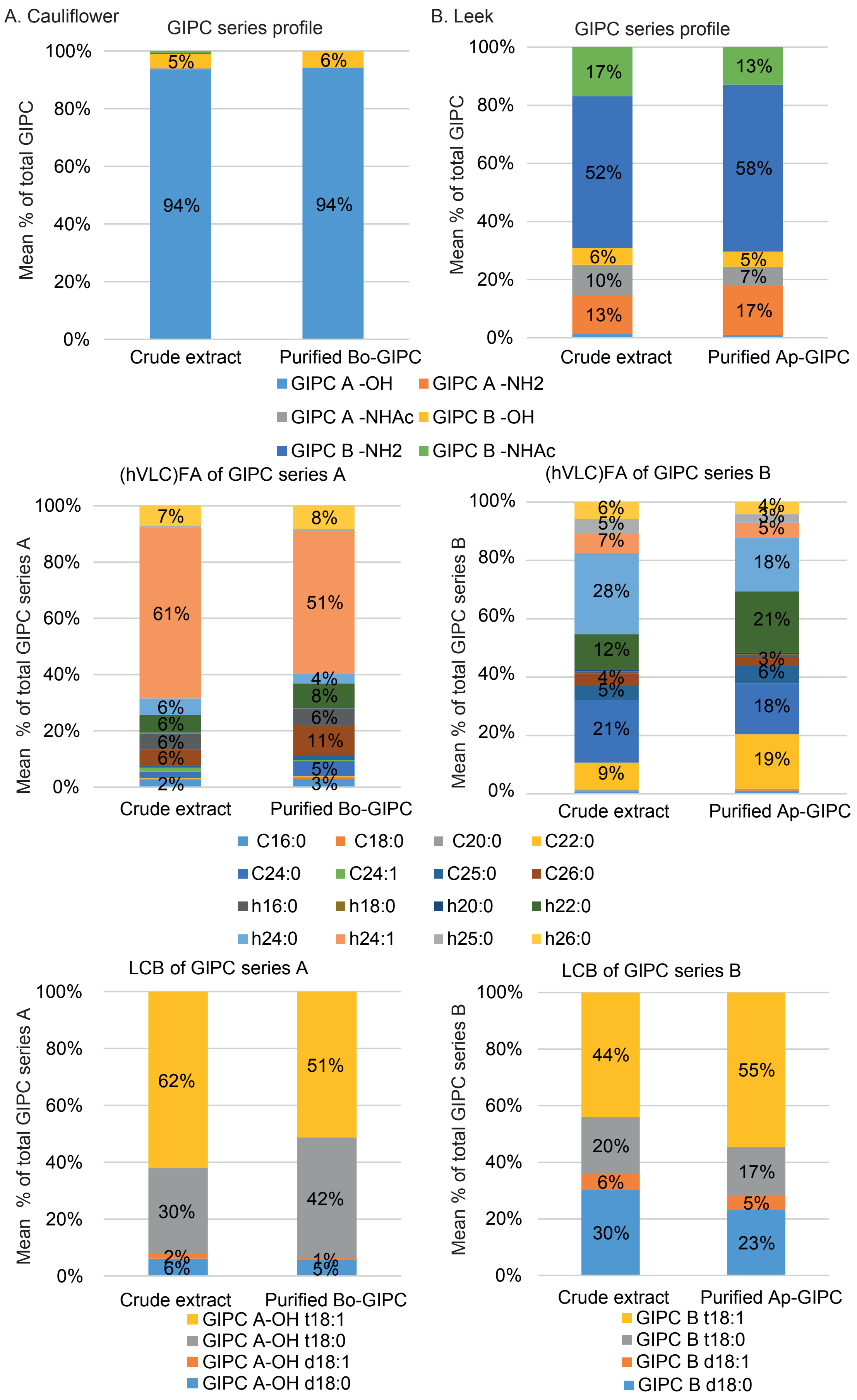

Supplemental data 6

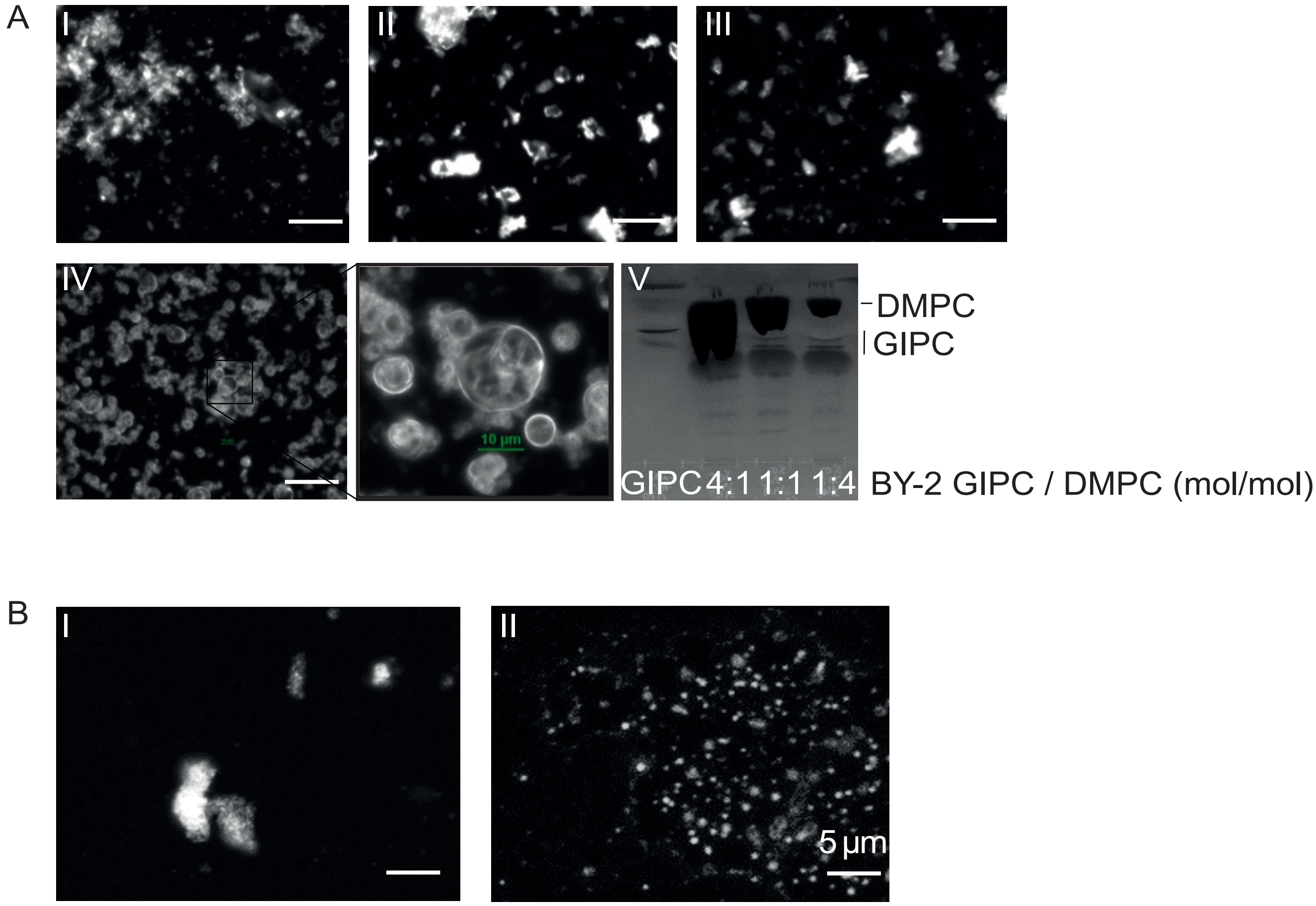

Supplement: Supplemental Table S1 [file mmc2.pdf]
